# Factors associated with the spatiotemporal distribution of dog rabies in Tunisia

**DOI:** 10.1371/journal.pntd.0012296

**Published:** 2024-08-05

**Authors:** Sana Kalthoum, Samia Mzoughi, Raja Gharbi, Monia Lachtar, Bassem Bel Haj Mohamed, Haikel Hajlaoui, Wiem Khalfaoui, Anissa Dhaouadi, Imed Ben Sliman, Chafik Ben Salah, Haykel Kessa, Hend Benkirane, Ahmed Jawher Fekih, Kahoula Barrak, Hajer Sayari, Chokri Bahloul, Thibaud Porphyre

**Affiliations:** 1 Centre National de veille zoosanitaire, Tunis, Tunisia; 2 Regional veterinary services, Kef, Tunisia; 3 Regional veterinary services, Sousse, Tunisia; 4 Regional veterinary services, Ariana, Tunisia; 5 Regional veterinary services, Mahdia, Tunisia; 6 Regional veterinary services, Bizerte, Tunisia; 7 Regional veterinary services, Beja, Tunisia; 8 Pasteur Institute of Tunis, Tunis, Tunisia; 9 Laboratoire de Biométrie et Biologie Évolutive, Université Claude Bernard Lyon 1, CNRS, VetAgro Sup, Marcy l’Étoile, France; Universidad Nacional Mayor de San Marcos, PERU

## Abstract

Despite continuous efforts of veterinary services to control rabies in dogs since 1982, rabies remains a cause of death in Tunisia, with more than five reported human cases in 2022. As little is known on the determinants of transmission of rabies in dogs, better understand which factors contribute to its spatial heterogeneity in Tunisia is critical for developing bespoke mitigation activities. In this context, we developed Bayesian Poisson mixed-effect spatio-temporal model upon all cases of rabid dogs reported in each delegation during the period from 2019 to 2021. The best fitting model highlighted the association between the risk of rabies and the mean average monthly temperature, the density of markets and the density of dogs in delegations. Interestingly, no relationship was found between intensity of vaccination in dogs and the risk of rabies. Our results provided insights into the spatio-temporal dynamics of dog rabies transmission and highlighted specific geographic locations where the risk of infection was high despite correction for associated explanatory variables. Such an improved understanding represent key information to design bespoke, cost-efficient, rabies prevention and control strategies to support veterinary services activities and policymaking.

## Author summary

Rabies, a deadly zoonotic disease, remains a public health concern in Tunisia despite ongoing prevention efforts. Our study, the first of its kind in Tunisia, aimed to analyze the spatiotemporal distribution of dog rabies and identify factors influencing its spread. Using a Bayesian approach, we examined the relationships between epidemiological, socio-economic, and environmental factors and the occurrence of rabies cases in different regions (delegations) of Tunisia. Our study revealed that variations in the risk of rabies were primarily associated with spatial effects rather than temporal trends. We found a negative association between average monthly temperature and dog rabies cases, consistent with decreased viral survival and dog activity in warmer temperatures. The risk of rabies cases had a non-linear relationship with dog density. We also found that dog rabies risk was positively associated with the density of slaughterhouses, indicating that these locations might contribute to interactions between infected and susceptible animals. Surprisingly, vaccination coverage did not show a significant association with rabies risk. Our findings contribute to the global goal of eradicating rabies by 2030 and underscore the importance of adapting strategies based on the unique factors influencing disease transmission in specific regions.

## Introduction

Rabies is a neglected diseases caused by a lyssavirus (*Rhabdoviridae* family) that kills more than 59,000 people annually worldwide [1]. Exposure to rabies usually occurs via a bite, mainly from dogs, and the virus is transmitted via the saliva of a rabid host [2]. Despite decades of prevention and control activities, rabies still poses a significant public health threat in many parts of the world, particularly in low-income countries where vaccination and control measures on dog populations are often limited [3]. This led the quadripartite (WHO, OIE, FAO and GARC) of the United Nations to call for action and lay a global strategic plan to “prevent human deaths from dog-transmitted rabies by 2030” [4].

Dog-mediated rabies transmission and spread are influenced by various factors, including dog population density, shared country borders, and socioeconomic conditions such as poverty [5]. Studies conducted by Morters et al. (2013), Yao et al. (2015), and Chikanya et al. (2021) [6–8] provided compelling evidence that socioeconomic factors, such as poverty, education levels and education access, are not just coincidental contributors to the dynamics of rabies transmission; rather, they are critical determinants that directly impact the effectiveness of disease control measures.

In Tunisia, rabies is endemic, and domestic dogs are the primary reservoir and vector of the disease. Effective vaccines and control programs have been available for the prevention and control of rabies in dogs and humans. However, there has been a recent increase in the number of rabies cases in both populations [9], indicating the necessity of developing novel bespoke strategies to effectively mitigate its spread. Particularly, the risk of dog-mediated rabies transmission exhibits a marked heterogeneous spatial distribution. Bouslama et al. (2020) identified six distinct clusters of dog rabies cases for the period from 2011 to 2016, which were concentrated in the Northern and center-eastern regions of Tunisia [10]. These findings were further supported by a subsequent analysis by Kalthoum et al. (2021) that examined reported cases over a four-year period from 2012 to 2015 [11]. Additionally, at the regional level, Hassine et al. (2021) detected significant clusters of rabies cases in the Governorate of Nabeul, located in the northern part of the country [12]. Although these findings emphasize the need for targeted interventions to effectively control and manage the local spread of rabies, the underlying factors driving these spatial patterns remain to be identified and understood.

To achieve the global target of eliminating rabies by 2030 in Tunisia, investigating which socioeconomic, ecological, environmental, and/or behavioral factors contribute to the spatial heterogeneity of rabies and its spatiotemporal dynamics is critical. Such an information is fundamental, as it would facilitate developing bespoke mitigation activities at local level to account for local specificities. In this context, Bayesian spatial and spatiotemporal random-effect models represent powerful tools for providing such an insight [13], notably by capturing the spatial and temporal structure of the data while adjusting for the effect of local variations in associated risk factors [14,15]. Here, using such a modelling framework, we aim to determine what factors may explain the spatial and spatio-temporal structure of rabies in Tunisia and identify areas that remain of higher risk of dog rabies.

## Materials and methods

### Ethical statement

The research conducted does not involve any form of direct manipulation, experimentation, or intervention with dogs. The study relies on historical surveillance data collected during routine disease monitoring activities and publicly published data. There was no deliberate involvement of live animals for research purposes.

### Study area

Tunisia is a northern African country, located at latitude 33°50’ N and longitude 9°24’E, bordering the Mediterranean Sea in the North and Est. Tunisia shared borders with Algeria in the West and Libya in the Est. The Tunisian area is estimated at 164,000 km^2^ and its human population was 11,803,588 inhabitants [16]. It consists of 24 governorates (level 1), each of which is further divided into 264 delegations (level 2). The shapefile used to map and conduct the analysis, which contains level two divisions of Tunisia was downloaded from https://gadm.org/download_country.html [17].

### Case and vaccination data

**Fig 1:**
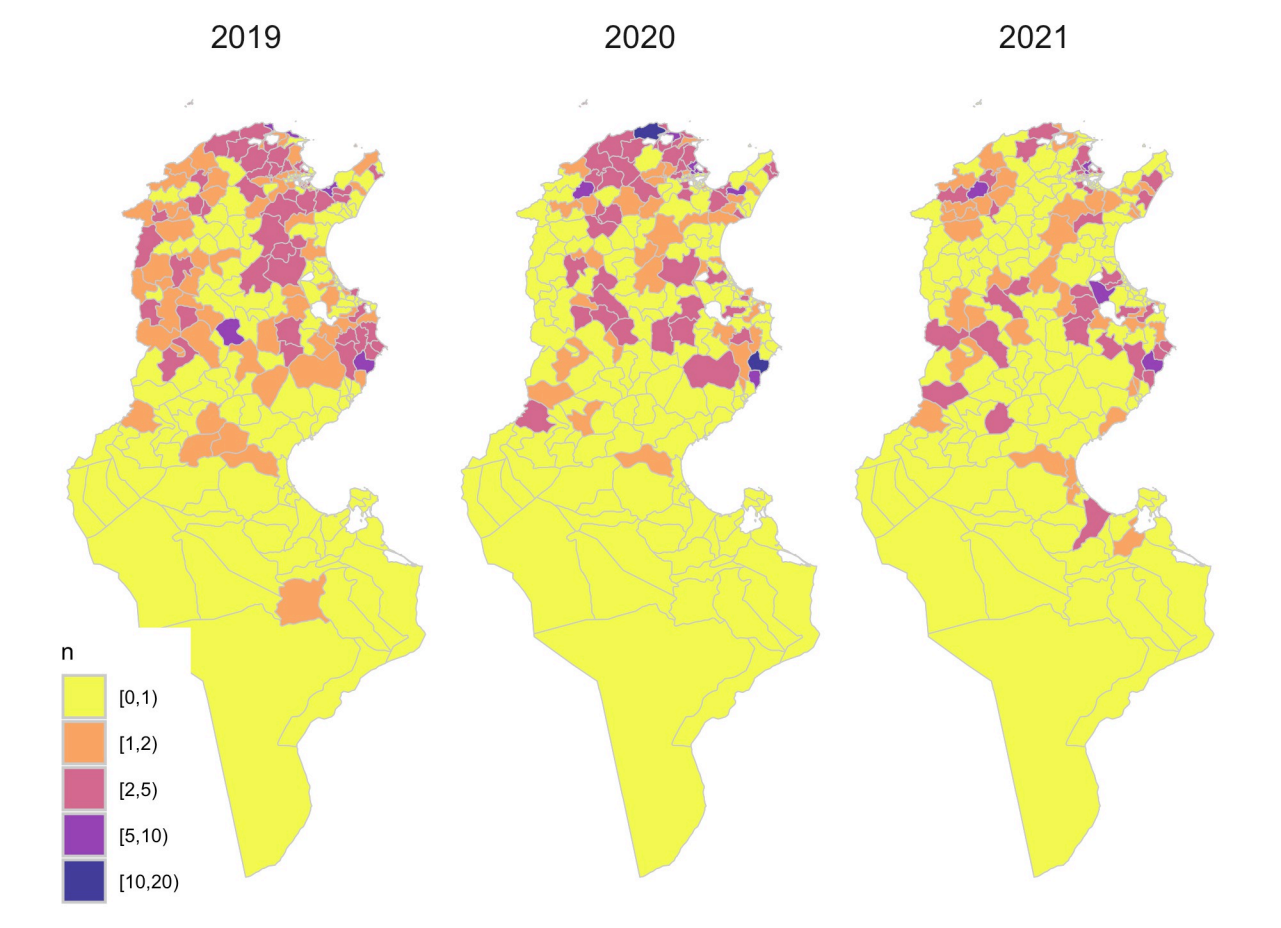
Spatial distribution of the number of reported cases of canine rabies in Tunisia, 2019–2021 (The shapefiles can be accessed directly at Tunisia Administrative Boundaries on GADM).

All confirmed cases of canine rabies reported during passive surveillance in Tunisia from January 1st, 2019, to December 31st, 2021, were extracted from the surveillance databases at the national and regional veterinary service offices (Fig 1). These cases were aggregated at the Delegation level for analysis. In Tunisia, dogs and other animal species displaying clinical signs of rabies are regularly sent to the Pasteur Institute of Tunis, which is the approved reference laboratory for rabies diagnosis in the country. The diagnostic of rabies is based on the Fluorescent Antibody Test, which detects the presence of rabies antigens in the brain tissue of tested animals. Virus isolation and real-time RT-PCR techniques utilizing the SYBR-Green fluorescent intercalant are also used for rabies diagnosis.

For the purposes of our analysis, data from Kerkennah islands were excluded. This is because we believe that Kerkennah islands may have unique characteristics or epidemiological factors that differ significantly from mainland Tunisia. These differences could potentially introduce biases or confounding factors that may affect the validity and generalizability of the results. In total, our analysis includes rabies surveillance data from 263 Delegations.

Vaccination coverage was measured by assessing the proportion of vaccinated dogs within the estimated dog population. It was calculated according to the following formulae: Vaccination Coverage (%) = (Total number of Vaccinated Dogs /Dog Population during the considered year) ×100.

The data were collected from records on the number of vaccinated dogs in each Delegation maintained by veterinary authorities at national and regional levels.

### Demographic and socio-economic data

**Fig 2:**
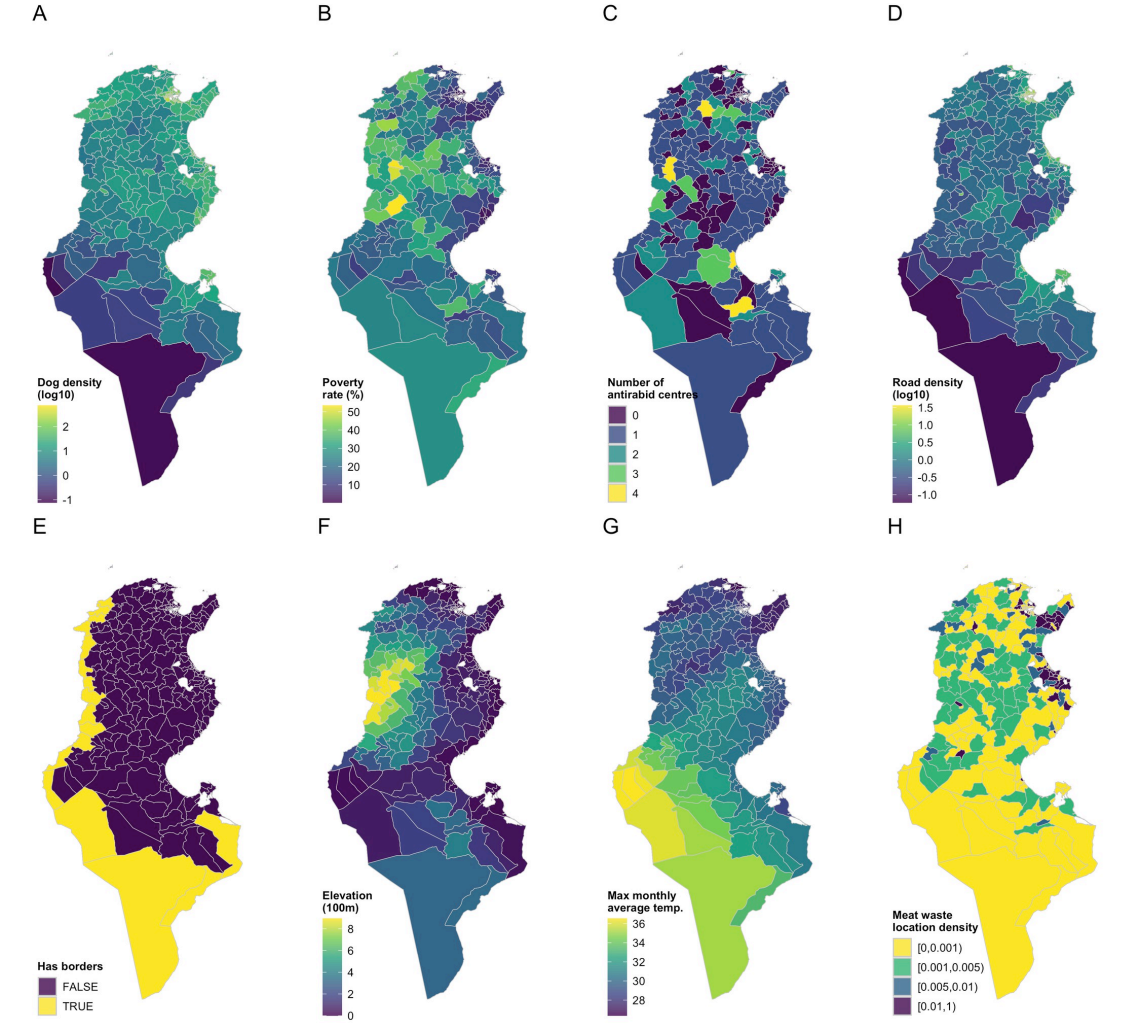
Spatial distribution of potential risk factors (The shapefiles can be accessed directly at Tunisia Administrative Boundaries on GADM).

For each of the 263 Delegations included in the analysis, information of putative risk factors was extracted from different freely available and governmental data sources. Population data were retrieved from the data sources provided by the National Institute of Statistics. Demographic and socio-economic variables included: human population density, dog population density, number of vaccinated dogs, human poverty rate, school dropout rate, road density, and number of anti-rabies centers (Table 1 and Fig 2).

**Table 1:** Epidemiologically important factors and variables hypothesized to modify the risk for dog-mediated rabies in Tunisia. The data time, sources, and administrative level at which the data were available are listed.

Variable	The administrative level of data availability	Year/s of data availability	Source of data
**Number of dog rabies cases**	Delegation (level 2)	2019–2021	National and regional veterinary services of Tunisia
**Number of rabies cases in other species**	Delegation (level 2)	2019–2021	National and regional veterinary services of Tunisia
**Human population**	Delegation (level 2)	2019–2021	National Statistical Institute of Tunis http://www.ins.tn/enquetes
**Dog population**	Delegation (level 2)	2019	Centre national de veille zoosanitaire
**Number of vaccinated dogs**	Delegation (level 2)	2019–2021	Regional veterinary services of Tunisia
**Human poverty rate**	Delegation (level 2)	2015	National Statistical institute of Tunis http://www.ins.tn
**School dropout rate**	Delegation (level 2)	2015	National Statistical institute of Tunis http://www.ins.tn
**Road density**	Delegation (level 2)	2023	Centre national de veille zoosanitaire
**Number of anti-rabies center**	Delegation (level 2)	1978–2023	http://www.rage.tn/Fr/

### Environmental data

Mean daily air temperature within a given month was extracted from the freely available CHELSA dataset version 2.1, which provides 1km-resolution climate data (https://chelsa-climate.org/downloads/). These temperature values were processed to calculate the overall mean temperature in each delegation, as well as the mean, minimum, and maximum monthly average temperature values. The average altitude of individual delegations was obtained from the 10km-resolution digital database of land and sea-floor elevations of the World (ETOPO5) (https://www.eea.europa.eu/data-and-maps/data/world-digital-elevation-model-etopo5).

In addition to temperature and elevation data, other variables such as borders, oued (river), number of slaughterhouses (red meat), number of slaughterhouses (white meat), and number of markets were considered. To assess their impact, densities of roads, oueds, slaughterhouses, and markets in each Delegation were computed.

Demographic and socio-economic variables (human population density, dog population density, number of vaccinated dogs, human poverty rate, school dropout rate, road density, and number of anti-rabies centers) were included in the model based on previous reports published in literature and on their relevance to the epidemiology of rabies and their potential to influence the transmission and spread of the disease [18,19]. Additionally, environmental factors were integrated recognizing their impact on rabies virus viability and transmission. Warmer temperatures have been associated with increased rabies activity, making it an important factor to consider in the analysis [20,21].

Other variables such as borders, oued (river), number of slaughterhouses (red and white meat), and number of markets were considered due to their potential impact on the movement and interaction of animals, which can influence the spread of rabies. These variables were included to capture the spatial and environmental factors that may contribute to the risk of rabies within each delegation.

To enable analysis, several categorical variables were transformed into binary indicators. The variable "borders" was incorporated as a binary indicator, with values of "0" denoting the non- sharing borders with neighbouring countries and "1" indicating the sharing borders with neighbouring. For instance, variables such as “slhr_cat” (slaughterhouses category) were categorized into different ranges, and binary indicators like “slhr_high” were created to represent specific categories (e.g., “[0.005,0.01)” and “[0.01,1)”). Similar transformations were applied to variables such as “slhw_cat” (slaughterhouses for white meat), “mkt_cat” (markets), and “mlw_cat” (markets for white meat). In our preprocessing steps, the categorization of variables related to the slaughterhouses and markets into different ranges was conducted to capture potential non-linear relationships and nuanced variations in the impact of these factors on the risk of reported rabies cases in dogs. The decision to create these multiple categories allows us to discern potential threshold effects or variations in the level of influence on rabies dynamics. If necessary, continuous variables were logarithmically transformed (Log10) to achieve normalization. Additionally, temperature variables were rescaled relative to the minimum temperature recorded. Finally, the elevation measures were scaled to represent changes per 100 meters of altitude increase. These preprocessing steps aimed to ensure the compatibility and appropriate scaling of variables for subsequent analysis.

### Statistical analysis

A Bayesian spatiotemporal Poisson regression model was developed to quantify the effect of factors influencing the number of canine rabies reported each year of the study period (2019–2021) in each of the 263 Tunisian Delegations of interest. All delegations (n = 263) were considered for the modelling process to provide a comprehensive overview of the rabies epidemiology across the entire study region. The number of reported cases in each year was adjusted for the frequency-dependence of the transmission by including an offset term corresponding to the expected number of reported cases of canine rabies for each Delegation and for each year of the study period. The expected number of cases that were reported in each year was considered as emerging from a homogenous process across the country and proportional to the surface area of each Delegation as suggested previously. The choice of a density-based approach for analysing the epidemiological situation of rabies involved calculating the number of cases per 100 km^2^. This approach was favoured over broad incidence indicators, like the total number of cases per year, which were considered overly generalized. The challenge with incidence indicators lay in the uncertainty surrounding population denominators for both dogs and humans, which can fluctuate over time, complicating the calculation of precise incidence rates. In response to this uncertainty, density-based indicators were adopted due to their ability to provide a more context-specific analysis, making them better suited for capturing localized variations and geographical patterns.

We used integrated nested Laplace approximations (INLA) [22] to conducted fast approximate Bayesian inference as detailed in Blangiardo et al (2013) [14]. Analyses were conducted using the *INLA* package [22] in the statistical environment R version 4.1.2 [23]. For our analysis, a series of models was first formulated to identify which model structure may best represent the underlying spatiotemporal structure of the data, assuming no influence of putative predictors. Each of the formulation include combination of function accounting for the delegation-specific effects *μ*_*i*_, year-specific effects *γ*_*t*_ and their interaction *δ*_*it*_. The selection of the most parsimonious model structure was based on three criteria: the Deviance Information Criteria (DIC) [24], the Watanabe-Akaike information criterion (WAIC) [25], and mean logarithmic scores [26]. These measures provide insights into the amount of variance explained by the model and its predictive quality. Smaller values of these criteria indicate better prediction performance. The null model, representing the spatio-temporal structure of the data, was formulated as shown in Table 2.

$$\eta_{i}=\log\left(\rho_{it}\right)=\beta_{0}+\mu_{i}+\phi_{t}+\delta_{it}$$

where the ratio *ρ*_*it*_ is the *standardized mortality ratio* (SMR, i.e. observed-to-expected rabies cases) for each Delegation *i* and in each year *t*; and *β*_0_ the intercept, quantifying the non-adjusted average rabies incidence rate in delegations. The component *μ*_*i*_ = *ν*_*i*_+*σ*_*i*_ is the random effects specific to each Delegation *i* and is the sum of the structured *σ*_*i*_ and unstructured *ν*_*i*_ random effects; whereas the components *ϕ*_*t*_ and *δ*_*it*_ represent the unstructured random effect specific to each year *t* and the interaction between space and time, respectively. Here, *δ*_*it*_ is assumed being the results of interaction between *ν*_*i*_ and *ϕ*_*t*_. Consequently, we assume no spatial and/or temporal structure on the interaction. Note that *ν*_*i*_ may allow for extra-Poisson variation (over dispersion) in the observed rabies counts, which may be caused by unknown, Delegation-specific and non-spatially structured confounding factors. However, we further evaluated presence of excess over dispersion and excess zeros in the data and the ability of the best fitting null Poisson model to account for them by fitting separate negative binomial and zero-inflated Poisson models, respectively. In both situations, the Poisson model was the preferred model in fitting the rabies data (ΔDIC > 60, Table 2).

**Table 2:** Selection of the model structure. For model structure considered to represent the spatio-temporal structure of the total number of cases of rabies in dogs per Delegation in Tunisia between 2019 and 2021. The Deviance information criterion (DIC), the changes in DIC between models (ΔDIC) the Watanabe-Akaike information criterion (WAIC) and the logarithmic score (LS) were computed for comparison.

Model	DIC	WAIC	ΔDIC	ΔWAIC	LS
*ν*_*i*_+*σ*_*i*_+*ϕ*_*t*_	1619.5	1659.3	50.5	82.9	1.21
*ν*_*i*_+*σ*_*i*_+*ϕ*_*t*_*+ξ*_*t*_	1620.1	1659.6	51.1	83.2	1.21
***ν***_***i***_ **+*σ***_***i***_ **+*ϕ***_***t***_ ***+δ***_***it***_	1569	1578.8	0	2.4	1.55
*ν*_*i*_+*σ*_*i*_+*δ*_*it*_	1569.4	1576.4	0.4	0	1.56
*ν*_*i*_+*ϕ*_*t*_*+ξ*_*t*_*+δ*_*it*_	1704.3	1740.4	135.3	164	3.43
*ν*_*i*_+*ϕ*_*t*_+*δ*_*it*_	1632.5	1623.9	63.5	47.5	4.63
*ν*_*i*_+*σ*_*i*_+*ϕ*_*t*_*+ξ*_*t*_+*δ*_*it*_	1569.8	1580.8	0.8	4.4	1.49

The component *μ*_*i*_ = *ν*_*i*_+*σ*_*i*_ is the random effects specific to each Delegation *I* and is the sum of the structured *σ*_*i*_ and unstructured *ν*_*i*_ random effects; whereas the component *γ*_*t*_ = *ϕ*_*t*_+*ξ*_*t*_ is the random effects specific to each year *t* and is the sum of represent the temporally structured and unstructured effect. The component *δ*_*it*_ represents the interaction between space and time. Model in bold indicates the best fitting model structure for the risk of rabies in dogs in Tunisia. The posterior estimate of the zero-probability parameter estimated from zero-inflated Poisson model was 0.1085 (95% Cr.I.: 0.0556; 0.1754), whereas the posterior estimate of the overdispersion parameter estimated from negative binomial model was 0.2767 (95% Cr.I.: 0.1315; 0.4808). The DIC of the Poisson model is much smaller than the DIC of both the model using zero-inflated model (ΔDIC = 43.9) and negative binomial model (ΔDIC = 60.5), indicating that there are limited zero excess and overdispersion in the data set, respectively; thus, indicating that the Poisson model is preferred in fitting the rabies data.

Once the best model structure was chosen, we examined the association of each putative risk factor separately with the risk of reported rabies cases in dogs, using uninformative priors to estimate the parameters. In this univariate analysis, variables showing an 80% credible interval (Cr.I.) of their posterior distribution that does not overlap 0 and that are not correlated to other variables (i.e., Kendal rank correlation coefficient, *τ*_*b*_, smaller than 0.7) were then taken forward to the multivariate analysis. We further evaluated the non-linear relationship of all associated continuous variables with the risk of canine rabies by adding random walk of the first order, *rw*1, and latent effect as a smooth term on the covariates of interest [27].

A stepwise elimination process was applied to retain associated variables. The DIC was used to compare model performance. Variables were considered significantly associated with the risk of reported rabies cases if their 95% credible interval of their posterior distribution does not overlap 0. In addition, the stability of all associations was checked by systematic removal of variables. Finally, it is worth noting that all tested putative risk factors were considered fixed for duration of the study period, assuming that no marked differences were observed during the three-year long study period.

Goodness of fit of final model was evaluated by comparing model-based predictions with observed number of rabies cases in dogs in each delegation and each year using Pearson’s product moment correlation coefficient, *r*. The uncertainty associated with the posterior means can also be mapped and provide useful information [14]. In particular, we were interested to identify which Delegations showed excess rabies risk through the whole study period and those that showed excess risk in given individual years. To do so, we plotted the spatial distribution of both posterior probabilities for the spatial random effect *p*(exp(*μ*_*i*_)>1|*y*) and for the spatiotemporal effect *p*(exp(*δ*_*it*_)>1|*y*). As defined in [14], an increased risk with a small level of associated uncertainty is indicated in villages with a spatial (or spatiotemporal) relative risk above one and an associated posterior probabilities above 0.95.

## Results

**Fig 3:**
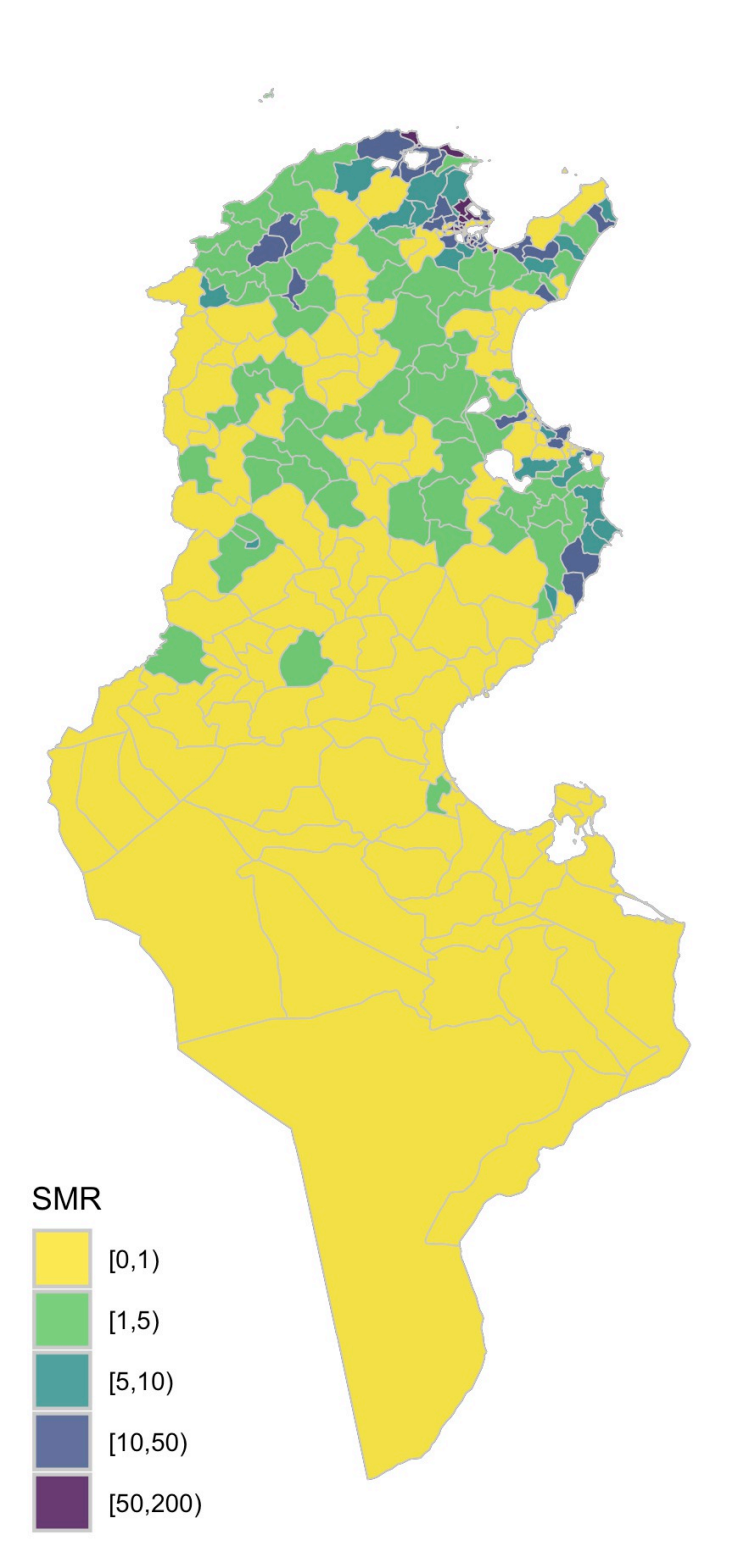
Spatial distribution of the aggregated standardized mortality ratio of canine rabies in Tunisia over the period 2019–2021 (The shapefiles can be accessed directly at Tunisia Administrative Boundaries on GADM).

Over the three years of our study period, 610 rabies cases were recorded in Tunisian dogs by national and regional veterinary services, which corresponds to a mean incidence of 28.2 reported cases per 100,000 dogs. Over the year, the number of reported cases progressively decreases from 33.6 cases per 100,000 dogs (a total of 229 cases) in 2019 to 19.9 cases per 100,000 dogs (or a total of 163 cases) in 2021. Overall, the number of cases of rabies reported in dogs varied between delegations (Fig 1), averaging around 2.3 cases reported within the study period and ranging from 0 to 22 cases. As such, the risk of rabies in dogs varied between delegations from 0 to 155 times more cases than what was expected with the largest (i.e.>5) standardized mortality ratio values centred on the Northern region between Tunis and Bizerte and the Eastern region between Sfax and Sousse (Fig 3).

**Fig 4:**
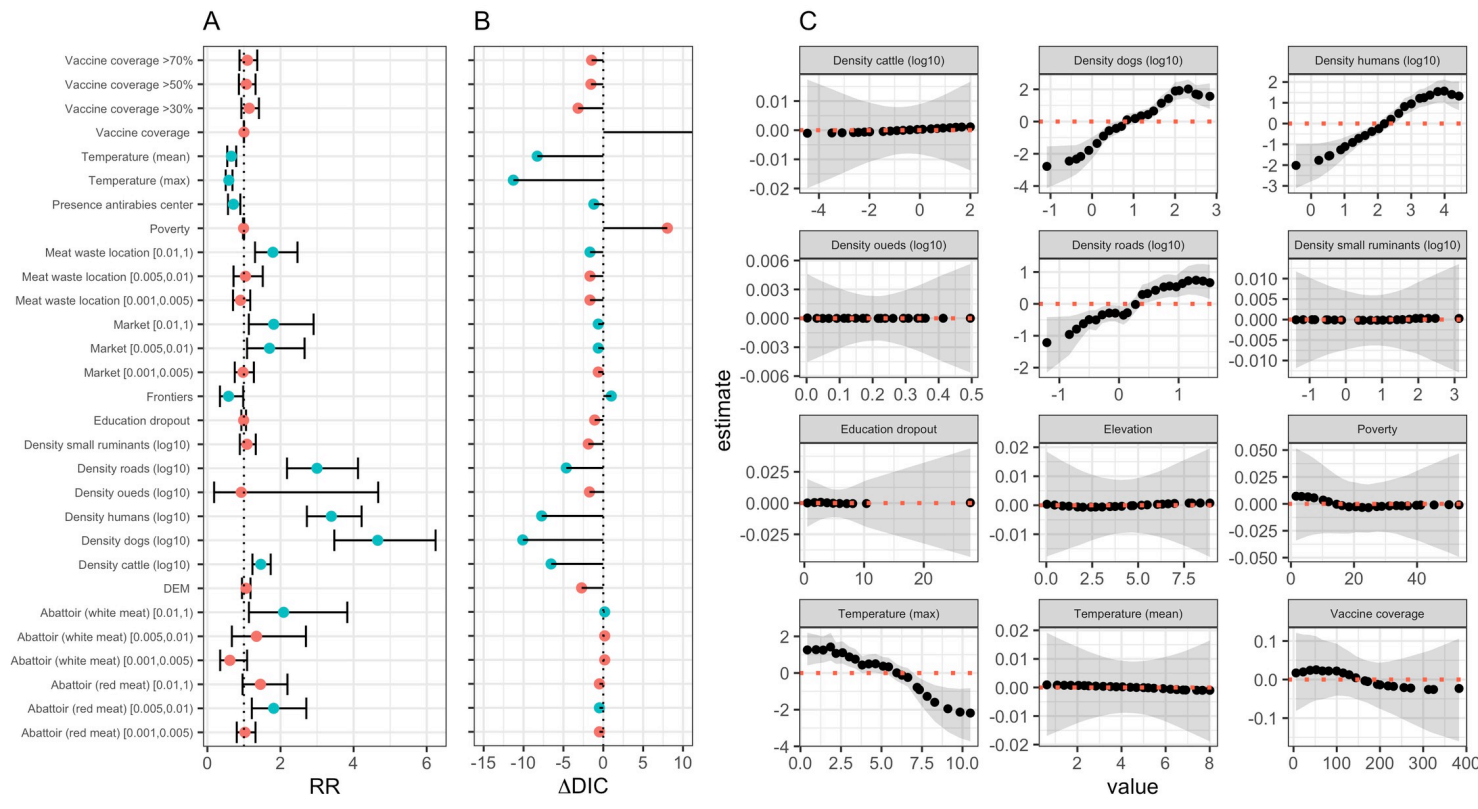
Relationship between considered risk factors and the risk of canine rabies in Tunisia.

Of the 21 linear risk factors tested, 12 were individually associated with the yearly risk of rabies in Delegations (Fig 4A). Among those, only six show some impact improving the null model (that is with a DIC smaller than the DIC of the null model and ΔDIC>2). These were the mean and maximum average monthly temperature in each Delegation and the log_10_-transformed density of dogs, humans and cattle as well as the log_10_-transformed density of road (Fig 4B). We further evaluated the shape of the relationship between continuous variables, such as dog, human, cattle and small ruminant (goat and sheep) population densities, altitude, vaccination coverage, density of roads and oueds, poverty and education dropout rates, and average monthly temperatures (mean and max) (Fig 4C). In addition to maximum monthly average temperature, only densities of dogs, humans and road were found associated with the risk of canine rabies in Tunisia. Not surprisingly, population density of dogs and human were highly correlated (Kendall τ_b_ = 0.77) and they were also correlated with the density of roads (τ_b_ = 0.59). Among the environmental variables, altitude was negatively correlated with the minimum average monthly temperature (τ_b_ = -0.77) but showed no relationship with both mean and maximum monthly average temperature (|τ_b_|<0.30). Here, the risk of rabies in Delegation was decreased by 34% (risk ratio (RR) = 0.66, 95% IC. 0.49–0.87) with each degree increase in mean monthly average temperature above 15°C, and by 41% (RR = 0.59, 95% IC. 0.46–0.75) with each degree increase in maximum monthly average temperature above 26°C.

**Fig 5:**
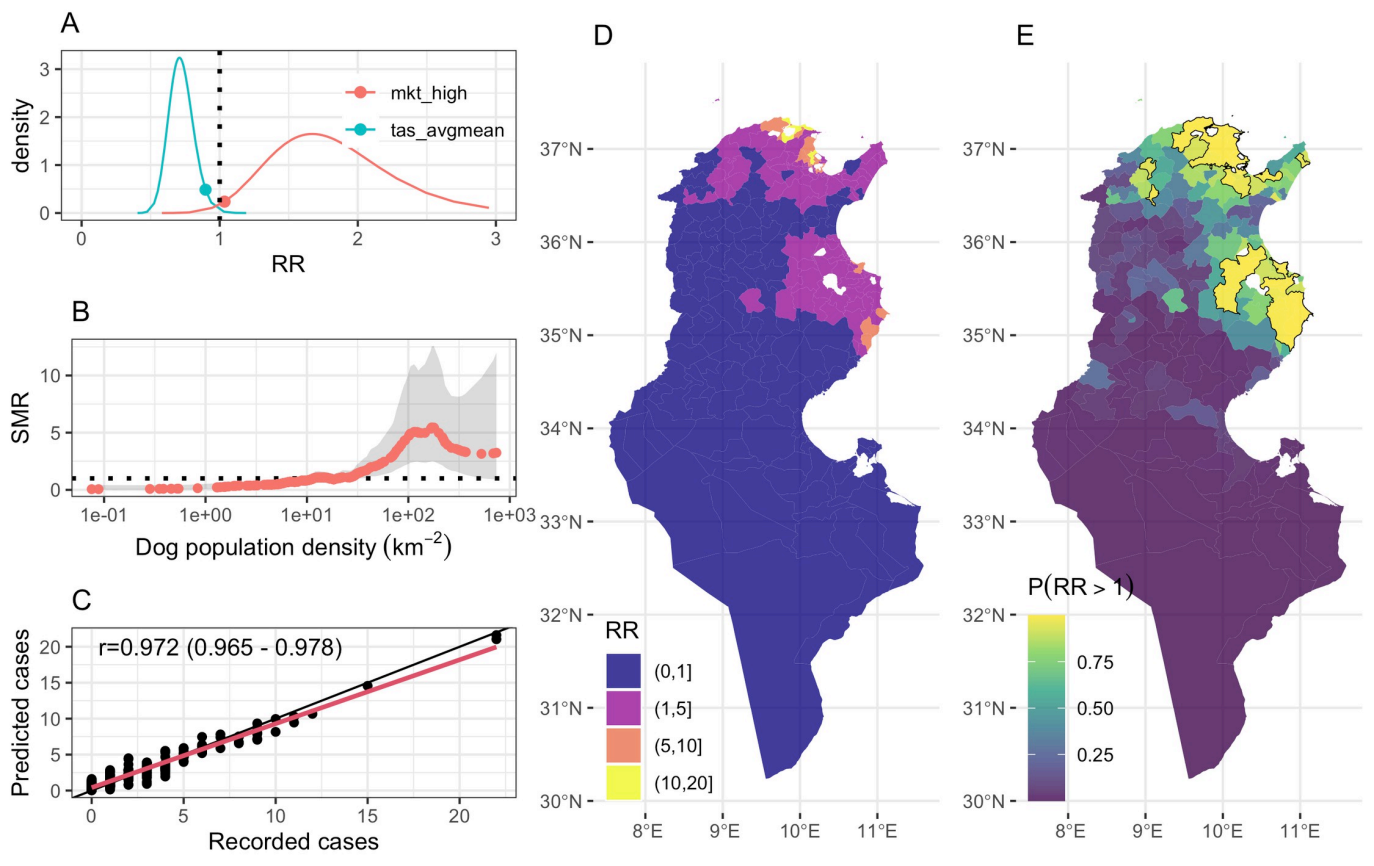
Final model outcomes and goodness of fit (The shapefiles can be accessed directly at Tunisia Administrative Boundaries on GADM).

No relationship was found between the risk of rabies and the socio-economic variables considered (poverty rate and education dropout rate). Similarly, whether assuming a linear or non-linear relationship, increasing the proportion of vaccinated dog did not show significant (in a Bayesian meaning of the word) relationship at the univariate level on the risk of canine rabies in Delegations. We further evaluated if delegations with a vaccine coverage of at least 30%, 50% or 70% of their dog population were less at risk of reporting rabies cases. However, no association was found with the Delegation-level of rabies risk during the study period. As such, information on vaccination coverage of dogs in Delegations were not considered further in our analysis. Similarly, no associations were found between the risk of reported rabies cases and the presence of anti-rabies centres in Delegations (Fig 5B).

Details of the final spatiotemporal model is shown in Table 3. As for the null model (Table 2), no evidence was found for the presence of excess zeros and over dispersion in the data set after accounting for explanatory variables (Table 3). In the final spatiotemporal model, the mean monthly average temperature was the most important factor associated with the risk of canine rabies in Tunisian Delegations (ΔDIC = 6.1, Table 3), showing the risk of rabies decreasing by 29% (RR = 0.71, 95% IC. 0.55–0.90) with each degree increase in mean monthly average temperature above 15°C. Although explaining little deviance in the model (ΔDIC<0.5), the risk of reporting rabies cases was further positively associated with the density of markets, increasing by 67% where there is more than 1 market per 200 km^-2^ (RR = 1.67, 95% IC. 1.04–1.69). Marginal posterior density distributions for both fixed effect variables are shown in Fig 5A.

**Table 3:** Model outcome.

	Median	2.5%	97.5%	RR (95%Cr.I.)	ΔDIC
**Fixed effects**					
**Intercept**	1.722	0.693	2.773		
**Density of market in Delegation (km** ^**-2**^ **)**					0.48
**[0,0.005)**	Ref.	-	-	-	
**[0.005,0.01)**	0.516	0.035	0.990	1.67 (1.0– - 2.69)	
**Adjusted Mean monthly average temperature (°C)**	-0.344	-0.593	-0.107	0.71 (0.5– - 0.90)	6.08
**Variance of the posterior distribution of nonlinear effect**					
**Dog population density (log10 km** ^**-2**^ **)**	0.987	0.367	2.560		3.24
**Variance of the posterior distribution of hyperparameters**					
**Spatial variance (*σ***_***i***_ **)**	1.947	1.329	3.020		59.7
**Delegation effect (*ν***_***i***_ **)**	0.00041	0.00012	0.0012		3.58
**Year effect (*ϕ***_***t***_ **)**	0.00005	0.00001	0.0002		0.14
**Spatio-temporal variance (*δ***_***it***_ **)**	0.241	0.123	0.417		48.7

RR = Risk ratio. ΔDIC = Changes in Deviance Information Criterion (DIC) due to the removal of a risk factor from the full model.

The risk ratio (RR) is calculated as the exponential of the posterior estimates and represents the proportional increase in risk per unit increase of the predictor. For each unit increases of the predictor, a value RR>1 indicates that the risk of canine rabies would increase, whereas a value RR<1 indicates that the risk would decrease.

The DIC indicates the performance of the model to explain the observed disease process, whereas ΔDIC shows how much each risk factor influences such performance. If ΔDIC = 0, this would indicate a variable with little influence in the full model to explain the observed disease process. In contrast, large values of ΔDIC would indicate the important variables in explaining the observed disease process.

Mean monthly average temperature (°C–—T_min_, where T_min_ = 15°C.

Variance of the posterior distribution of the structured spatial process.

Variance of the posterior distribution of the spatio-temporal process.

Variance of the posterior distribution of the unstructured spatial process.

These variance measures indicate the degree of variability in the disease process that is not explained by the included predictors.

The posterior estimate of the zero-probability parameter estimated from zero-inflated Poisson model was 0.0007 (95% Cr.I.: 0.0006; 0.0008), whereas the posterior estimate of the over dispersion parameter estimated from negative binomial model was 0.2801 (95% Cr.I.: 0.1351; 0.4822). The DIC of the final Poisson model is much smaller than the DIC of models of similar structure but using either zero-inflated model (ΔDIC = 984) or negative binomial model (ΔDIC = 58.0), indicating that there are limited zero excess and over dispersion in the data set, respectively; thus, indicating that the Poisson model is preferred in fitting the rabies data.

The risk of canine rabies was further associated with the density of dogs in delegations, showing a non-linear relationship (Fig 5B); with a lower risk of reporting rabies in dogs than expected when dog population density is lower than 6.4 dogs km^-2^. In contrast, delegations with a dog population at a density greater than 33.5 km^-2^ showed a greater risk of reporting cases than expected. It is worth noting that the risk of rabies would progressively increase with increasing dog density until it reaches its peak (RR = 5.32, 95% Cr.I.: 2.54–11.68) in Delegations showing a dog density of 170 dogs km^-2^. For density greater than 170 dogs km^-2^, the risk appears to stabilise around RR = 3.4, though tends to be very uncertain.

We assessed the explanatory performance of the final spatiotemporal model by examining how the model predictions agree with the observations (Fig 5C) and calculating the Pearson’s correlation coefficient between observations and predictions. The Pearson’s correlation coefficient was 0.97 (95% Cr.I.: 0.96–0.98, p<0.0001), indicating a high concordance of the model inferences with observations. However, such a high concordance is explained by the spatial and spatiotemporal effects (ΔDIC>100, Table 3), rather than the explanatory variables.

Looking at the spatial random process only (Fig 5D), it is clear that the spatial structure relating to the risk of reported canine rabies in Tunisia (Figs 1 and 3) has been only partially explained by the three significant explanatory variables, particularly at the South and West of the country. However, the posterior probability of the excess risk, *p*(exp(*μ*_*i*_)>1|*y*)>0.95, (Fig 5E) reveals there were three areas (solid thick contour) where the risk of rabies reports was significantly high but was not explained by the significant explanatory variables. These areas encompass Delegations between Tunis and Bizerte (in the North); those between Sfax and Sousse (in the East); and around Kairouan (the eastern region between the two salt lakes). The presence of areas with higher risk of rabies indicates that additional factors may take place leading to the disease to circulate (or being reported) at a greater rate than other areas.

**Fig 6:**
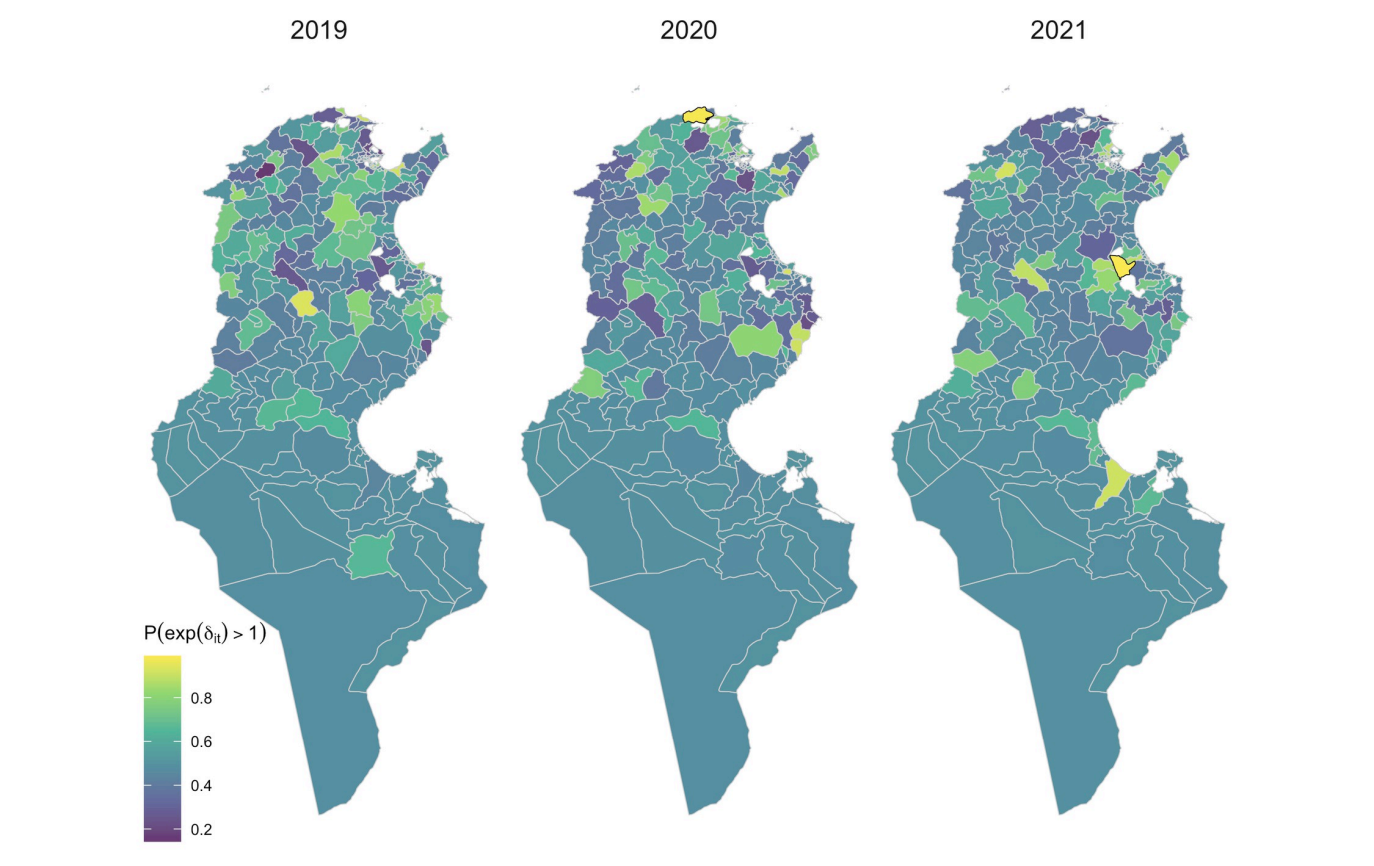
Distribution of the Delegation-specific posterior probability of the spatiotemporal random effect (The shapefiles can be accessed directly at Tunisia Administrative Boundaries on GADM).

The location of Delegations showing significant higher residual spatiotemporal risk, as indicated by posterior probabilities *p*(exp(*δ*_*it*_)>1|*y*)>0.95, are shown in Fig 6, represent additional risk to those emerging from the different explanatory variables and Delegation-specific spatial effects. These results reveal that Bizerte (in 2020) and Sidi El Heni (in 2021) represent hotspots of infection that occurred in specific years.

## Discussion

Despite ongoing rabies prevention programs, Tunisia have experienced an increase of rabies cases since 2012 in the north and the center of the country. To achieve zero rabies cases by 2030, it is crucial to understand the factors that contribute to its spread and persistence in order to implement effective prevention and control measures. Here, we analyzed all cases of rabies in dogs reported in Tunisia by taking into account both the spatial and temporal aspects of the disease [28]. This provides better understanding of the trends and variability in spatiotemporal patterns of geographical variation in rabies risk and the identification of clusters of cases or high-risk areas. Additionally, the model enables the exploration of relationships between potential risk factors and the risk of reported canine rabies, while controlling for confounding effects and accounting for spatiotemporal variations. By a Bayesian approach, the model can also account for uncertainty in the estimates and provide credible intervals for the results, thereby obtaining a more robust assessment of the associations between risk factors and canine rabies [29]. Furthermore, the incorporation of spatial information helps identify areas where targeted interventions can be implemented to effectively control the disease.

By incorporating socioeconomic, environmental, and epidemiological factors, the model allowed us to examine the impact of these variables on the occurrence of dog rabies cases while accounting for the underlying spatiotemporal dynamics [30]. To the best of our knowledge, this study is the first to test the association of epidemiological, socio-economic and environmental risk factors with rabies cases in Tunisia.

While no significant time effect was observed during the three-year study period from 2019 to 2021, it’s important to note that variations in the risk of rabies in dogs were primarily associated with spatial effects. Peaks in rabies risk were specifically observed in the northern and central regions of Tunisia in specific years. This is not surprising since clusters in the north and the center of Tunisia have been already highlighted in previous study on rabies in Tunisia [11]. Spatial variation of the risk factors can be potential reasons for the different risk among delegations. For example, areas with more abundant wildlife reservoirs of the virus or higher proportion of stray dogs may exhibit increased rabies risk. It was also demonstrated that areas with more extensive human-dog contact, the risk of rabies transmission may be amplified. Historical patterns of rabies incidence in a region can also influence current risk. Areas with a history of rabies may be more prone to recurring outbreaks due to the presence of the virus in the local ecosystem. Local customs and cultural practices can impact rabies risk. For instance, certain areas may have a tradition of keeping dogs as pets, while others may have a higher prevalence of free-roaming or feral dogs, affecting the dynamics of rabies transmission.

However, our study uses retrospective rabies data collected by veterinary services. As such, our findings should be interpreted with caution, as two important limitations are associated with the use of passive surveillance data. Firstly, it is crucial to acknowledge that the surveillance data used in this study primarily consisted of confirmed rabies cases that exhibited visible signs of the disease, leading to their identification and inclusion for testing. This approach introduces the potential for a population bias, as the sampled dogs with evident symptoms may not accurately represent the entire dog population. Focusing on symptomatic cases may result in an overrepresentation of specific subgroups within the dog population, potentially introducing bias into the findings.

Secondly, it is worth noting that, despite employing zero-inflated models, the model fit was not significantly improved. This may suggest that, if there are missing cases, the distribution of these missing cases appears to be relatively consistent across Tunisia, reflecting a level of homogeneity. Additionally, it is important to consider that the samples collected for testing may have originated primarily from areas in close proximity to the testing laboratory. This spatial bias in the sampling locations should be taken into account when interpreting the results, as it could affect the generalizability of the findings to other geographic regions in Tunisia.

Nevertheless, our study revealed a negative association between the average monthly temperature and the dog rabies cases in each Delegation. The association between temperature and rabies risk is consistent with previous studies [31,20]. This is likely due to the influence of temperature on the survival and activity of the rabies virus and dogs. Warmer temperatures may also result in altered patterns of dog activity, potentially leading to lower transmission rates. The study by Rabaiotti et al. in 2021, suggests that dogs, like many animals, may exhibit changes in behavior in response to temperature variations. In warmer climates, dogs might reduce their activity levels, alter their movement patterns, or seek shelter more frequently. These behavioral changes could contribute to a decreased likelihood of encounters between rabid and susceptible animals, subsequently influencing transmission dynamics [32]. Our findings are inconsistent with results reported in other regions. It has been observed that higher temperatures are associated with an increased risk of rabies. Months with high temperatures can likely be attributed to increased dog activity and consequently a rise in the number of dog bites [33]. The reason for this difference could be due to various factors, including regional variations, local conditions, and specific research findings.

The density of dogs was found to have a non-linear relationship with the risk of rabies in Tunisian delegations. At lower densities (below 6.4 dogs/km^2^), the risk of reporting rabies cases was lower than expected. However, as dog density increased beyond 33.5 dogs/km^2^, the risk of rabies cases also increased. The peak risk was observed at a dog density of 170 dogs/km^2^, where the risk was 5.32 times higher than expected. This finding suggests that there may be a threshold density beyond which the risk of rabies transmission becomes significantly important. Higher dog densities can facilitate closer contact and interactions between infected and susceptible animals, leading to increased transmission rates [34,35]. Invasion thresholds, observed in numerous studies such as those conducted by Steck and Wandeler in 1980, Beran and Frith in 1988, and Cleaveland and Dye in 1995, represent a critical concept in disease ecology. These thresholds indicate the minimum host population density required for a pathogen to establish and spread effectively. They depend on various factors, including pathogen characteristics, host susceptibility, and environmental conditions. Invasion thresholds have significant implications for wildlife and domestic animal populations, guiding disease management efforts and resource allocation. Understanding these thresholds is essential for predicting disease dynamics, especially in zoonotic diseases, and for implementing targeted control strategies in affected regions [36–38]. These thresholds indicate a transition from sporadic disease occurrences at lower densities to persistent transmission at higher densities, specifically observed in the case of canine rabies. However, it is important to note that establishing a definitive relationship between host density and disease incidence based only on these studies is not reasonable [38].

In this study, we found that the density of the slaughterhouses was positively associated with the risk of dog rabies cases in Tunisian delegations. Slaughterhouses often attract large numbers of dogs, increasing the likelihood of interactions between infected and susceptible animals [39]. In this situation, drawing definitive conclusions on the relationship between host dog density and the incidence of rabies often requires considering a broader set of variables and conducting more comprehensive, location-specific studies. This is especially relevant in complex disease systems like rabies transmission, which involve interactions between animals, humans, and the environment.

It is important to note that this study focused on risk factors at the delegation level and did not establish substantial associations with specific socioeconomic indicators such as poverty rate and education dropout rate. Our study’s focus on the delegation level might not have been fine-grained enough to capture intricate relationships between socioeconomic variables and the incidence of rabies. The impact of these factors can vary considerably within a single delegation, making it challenging to identify these effects when analyzing larger geographic units. Our result is similar to previous research conducted by Mogano et al. in 2022, in which they also did not highlight significant correlations between poverty and rabies [40]. However, these findings should be interpreted with caution as other studies have identified poverty as important determinants of rabies risk [5, 41].

In contrast to the well-established importance of vaccination in preventing rabies infection, our study did not identify vaccination as a significant risk factor for rabies, consistently with the study of Arias-Orozco et al. (2018) [5]. We acknowledge that the quality and granularity of the data used in the study may provide insufficient details on vaccination status or increase the background underreporting of rabies cases, and thereby affect our ability to establish a significant association. However, given the proven benefit of vaccination on controlling rabies in dogs [42], we were expecting a marked relationship between vaccination coverage and the risk of reporting rabies in Delegations. However, the unclear findings may be attributed to several factors. Firstly, it is plausible that vaccination coverage within the study population was sufficiently high, resulting in limited variability in vaccination status and, consequently, diminishing the statistical power to detect a significant association. Additionally, there could be other influential factors at play, such as vaccine effectiveness or variations in exposure to the rabies virus across diverse geographic regions. Furthermore, it is crucial to acknowledge the spatial scale of our analysis. The current delegation-level analysis, while informative, may not capture the finer variations in vaccination effects that could manifest at a more localized scale. Vaccination impacts may be more apparent when examined with higher resolution, such as at the level of individual communities or neighborhoods. This consideration is particularly relevant when assessing the effectiveness of vaccination strategies in diverse regions with varying risk profiles. This result can also be attributed to the methodology used in calculating vaccination coverage. This method relies on the number of estimated dogs, which does not account for puppies and new introductions. This limitation in data collection might have masked the true impact of vaccination on rabies incidence.

Finally, we identified areas with higher residual spatial and spatiotemporal risk that were not fully explained by the significant explanatory variables. These areas, including delegations between Tunis and Bizerte, between Sfax and Sousse, and around Kairouan, indicate the presence of additional factors influencing the transmission and reporting of rabies. While our analysis adjusted the risk of rabies for the effect of putative explanatory variables, we acknowledge that the role of a few key factors were not evaluated. These factors include post-exposure prophylaxis (PEP), the proportion of free-roaming dogs in delegations, the presence and size of public landfills, the presence and intensity of culling interventions targeting dogs, the local density of veterinary clinics, and the influence of wildlife species. Recognizing these factors as potential contributors to rabies risk [21,43,44], their specific relevance to the identified high-risk areas necessitates more in-depth investigation. Importantly, we emphasize that these factors are not assumed to be universally applicable or exclusive to specific delegations but may exhibit varied significance across regions. Therefore, future research is needed to explore the impact of these factors and their interactions within the socio-ecological context of the identified high residual risk areas. Local factors, such as dog movement patterns, cultural practices, and human-dog interactions, which may contribute to the persistence and circulation of the disease in these regions, must be studied. Such nuanced insights will be pivotal in informing the design of targeted interventions and effective control strategies tailored to the unique circumstances of each specific area, ultimately advancing our collective efforts towards rabies eradication in Tunisia.

## Conclusion

The present study aims to gain valuable insights into the factors associated with the risk of reporting rabid dogs through a spatiotemporal model at the delegation level. Through our analysis, we identified areas at higher risk of dog rabies, with population densities of dogs emerging as an important contributor to the dynamics of reported rabies cases. Additionally, we observed that temperature thresholds play a role in the risk of reporting rabid dogs, with higher monthly average temperatures associated with a decreased risk of rabies transmission. However, the absence of a significant association between several factors and the occurrence of reported rabies underscores the need for further research. Future studies should consider exploring additional potential risk factors, such as post-exposure prophylaxis, free-roaming dog populations, land use practices, and wildlife species. It is crucial to note that our study primarily addresses the risk of dog rabies, and our findings have important implications for designing targeted interventions and control measures to effectively mitigate this zoonotic disease’s risk. These efforts contribute to the global initiatives aimed at eradicating this deadly zoonotic disease by 2030.

## Supporting information

S1 Table Dataset. (XLSX)
